# Heterogeneous Reactions of Phenol on Different Components of Mineral Dust Aerosol: Formation of Oxidized Organic and Nitro-Phenolic Compounds

**DOI:** 10.1021/acsestair.3c00042

**Published:** 2024-02-23

**Authors:** Eshani Hettiarachchi, Vicki H. Grassian

**Affiliations:** Department of Chemistry and Biochemistry, University of California San Diego, 9500 Gilman Drive, La Jolla, California 92093, United States

**Keywords:** phenol, iron oxide, nitro-phenolic compounds, mineral dust, heterogeneous reactions

## Abstract

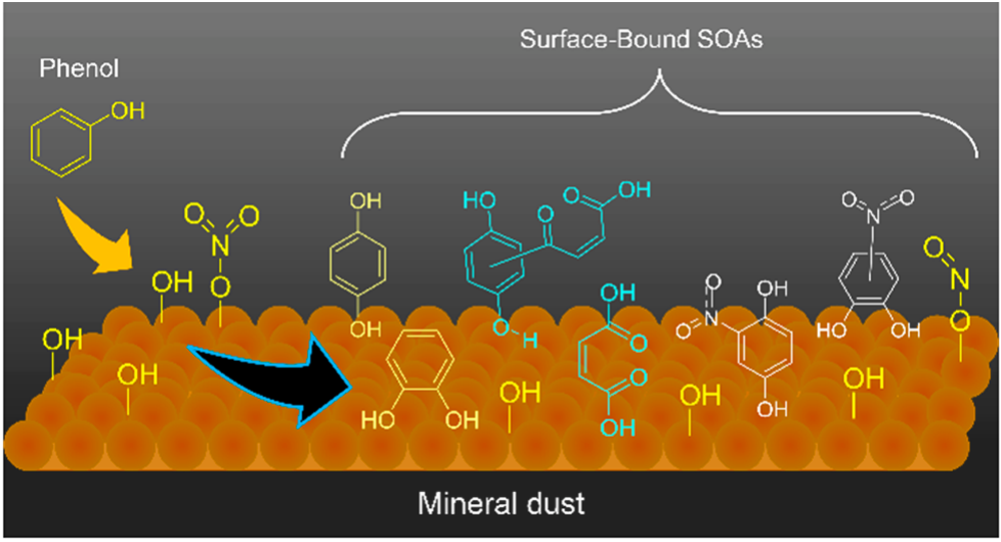

Phenol, a common semi-volatile compound associated with different emissions including from plants and biomass burning, as well as anthropogenic emissions and its derivatives, are important components of secondary organic aerosols (SOAs). Gas and aqueous phase reactions of phenol, in the presence of photochemical drivers, are fairly well understood. However, despite observations showing aromatic content within SOA size and mass increases during dust episodes, the heterogeneous reactions of phenol with mineral dusts are poorly understood. In the current study, surface reactions of phenol at the gas/solid interface with different components of mineral dust including SiO_2_, α-Fe_2_O_3_, and TiO_2_ have been investigated. Whereas reversible surface adsorption of phenol occurs on SiO_2_ surfaces, for both α-Fe_2_O_3_ and TiO_2_ surfaces, phenol reacts to form a wide range of OH substituted aromatic products. For α-Fe_2_O_3_ surfaces that have been nitrated by gas-phase reactions of nitric acid prior to exposure to phenol, unique compounds form on the surface including nitro-phenolic compounds. Moreover, additional surface chemistry was observed when adsorbed nitro-phenolic products were exposed to gas-phase SO_2_ as a result of the formation of adsorbed nitrite from nitrate redox chemistry with adsorbed SO_2_. Overall, this study reveals the extensive chemistry as well as the complexity of reactions of prevalent organic compounds leading to the formation of SOA on mineral surfaces.

## Abstract 2

Heterogeneous reactions of phenol, a common aromatic compound, on mineral dust aerosol surfaces show a number of products including different phenol oxidation products and nitrophenols, in the presence of adsorbed nitrate.

## Introduction

Secondary organic aerosols (SOAs) are ubiquitous in the atmosphere.^1−8^ Phenol (C_6_H_6_O) and its derivatives such as nitrophenols and catechol are compounds found within SOAs and represent a structurally unique mixture of compounds found in polluted environments.^9−18^ These compounds are detrimental to human health.^19−21^ Apart from health concerns, phenolic compounds are important light-absorbing species contributing to the atmospheric brown carbon formation.^22−24^ Phenol and OH substituted phenols such as catechol and guaiacol are key components in biomass burning.^14,15,25^ Vehicle exhausts,^26^ pesticide degradation, industries such as resin processing, coal production, and combustion^27,28^ can emit phenol and phenolic compounds into the environment.^18^ Phenol concentration in ambient air is 0.03–44 ppb,^18,29^ whereas the gas-phase concentration of nitrophenols is 0.02–56 ppt.^30^

Phenol and its derivatives in the atmosphere undergo photochemical reactions with OH radicals to produce various oxidized compounds in the gas phase and/or aqueous phase.^14,30−36^ These include catechol, hydroquinone, and phenoxy radical, which all can undergo further reactions.^14,37^ Further oxidation of catechol and hydroquinone in the presence of OH radicals leads to aromatic ring opening to produce aliphatic carboxylic acids. Ring opening of catechol leads to the formation of *but***-**2,4-dien-1,6-dicarboxylic acid (muconic acid), whereas hydroquinone forms maleic and oxalic acids. Under strong oxidizing conditions, the C_6_ dicarboxylic acid formed from catechol further oxidizes to maleic and oxalic acids.^14^ In some cases, the phenoxy radical can be involved in radical mediated reactions yielding oligomers and radical coupling.^14,34^ Phenol also reacts with nitrogen oxides including NO_2_ and NO_3_ along with OH radical to form various nitrophenols, among which 2-nitrophenol and 2,4-dinitrophenol are the most common.^33^ Nitrophenols formation from catechol can also occur in aqueous environments in the presence of NO_2_ or HNO_3_.^14,18,34,38,39^

Although phenol reactions in the aqueous and gas phases have been well studied, reactions of phenol on mineral surfaces are scarce. The chemistry of other aromatic compounds, including several related compounds, have been previously investigated.^40−49^ In one study, reactions of guaiacol on Fe-containing clay minerals were shown to contribute to brown carbon formation under varying relative humidity levels. Fe(III) has been shown to oxidize and catalyze the polymerization of guaiacol. Moreover, higher RH accelerated the polymerization reaction.^40^ Ozonolysis of adsorbed 4-propyl-guaiacol and catechol on NaCl and α-Al_2_O_3_ surfaces has also been shown in the formation of aromatic ring opening products.^41,42^ In another study, formation of maleic acid from chlorobenzene on γ-Al_2_O_3_^43^ and a significant degradation on α-Fe_2_O_3_^44^ under photochemical conditions were observed. Aromatic compounds such as phenol and benzene have been shown to undergo hydroxylation in the presence of TiO_2_ and iron oxides when H_2_O_2_ is present as the oxidizing agent,^45,46^ whereas phenol in water is shown to undergo selective photo-oxidation in the presence of TiO_2_.^47^ Studies conducted with pyridine, a model PAH, and NO_2_ showed the heterogeneous nitration rates are accelerated on mineral dust, particularly on kaolinite.^48^ Another study conducted on heterogeneous reactions of toluene with NO_2_ on α-Fe_2_O_3_ has reported enhanced ON formation with decreasing temperature, whereas addition of O_3_ enhances ON formation with increasing temperature, indicating the various heterogeneous reaction mechanisms at play.^49^

Overall, there is increasing recognition of the impact of mineral dust aerosol, a reactive and abundant aerosol,^50^ on SOAs concentration and aerosol size distributions.^51−54^ For an example, during a dust event from the Gobi Desert, an increased concentration of nitro-phenolic compounds in the size range of (3.2–5.6 μm) was seen compared to nondust events. This observation was ascribed to the vast surface area provided by the dust facilitating the heterogeneous formation of these SOAs and reactions of HNO_3_, NO_2_, and other NO_*x*_ with the dust surfaces.^16,51^ In another study, enhanced SOA formation during dust episodes was attributed primarily to photochemical reactions.^54^ While mineral dust carried by these dust episodes clearly provide a surface for these heterogeneous reactions to occur, the specific mineralogy of the dust provides different reaction pathways that are not delineated by just surface area and generalized reaction schemes. Thus, it is important to parse out mineral-specific heterogeneous and multiphase reactions. Despite the numerous observations aforementioned indicating the active participation of surfaces on reactions, heterogeneous formation pathways of oxidized organics important for SOA formation from aromatic alcohols such as phenol in the presence of mineral dust are poorly understood.

In this study, reactions of phenol on α-Fe_2_O_3_, TiO_2_, and SiO_2_ were studied at 298 K under dark conditions. In addition, these reactions of phenol with pre-nitrated oxide surfaces forming nitro-phenolic and oxidized aromatic organic compounds in the absence of water and photochemical drivers were studied. Surface-adsorbed products with gas-phase SO_2_ were studied to determine if organosulfur compounds could be formed. Both α-Fe_2_O_3_ and TiO_2_ are major reactive components of mineral dust, whereas SiO_2_ is a non-reactive component.^50,55^ These mineral surfaces can interact with gas-phase NO_2_, HNO_3_, and SO_2_ emitted to the atmosphere via both natural and anthropogenic sources,^5,48,56−60^ leading to the formation of nitrated- or sulfated-mineral-surfaces, respectively.^58,60−64^ Adsorption of these inorganic species on mineral surfaces has been widely studied.^61−63,65−67^ Adsorption of NO_2_/HNO_3_ on mineral surfaces yield surface adsorbed nitrates and nitrites, thereby producing a surface for phenol to interact with and facilitating the formation of organonitrate (ON) compounds. In environments where SO_2_(*g*) is present, these surface-bound various SOAs can further react on the mineral surfaces. For these studies, both Fourier transform infrared (FTIR) spectroscopy and high-resolution mass spectrometry (HRMS) were used to better understand the chemistry of phenol on environmentally relevant surfaces. For additional thermodynamic and molecular vibrational frequency calculations, Spartan ’20 Version 1.1.4 (Wavefunction Inc) was utilized.

## Materials and Methods

### In Situ Monitoring of Surface Adsorption and Reaction with Transmission FTIR Spectroscopy

Transmission FTIR spectroscopy was used to monitor reactions on mineral surfaces at 298 ± 1 K. Additional details of this system have been previously described.^68−73^ Oxide particles (SiO_2_, Aerosil OX50, α-Fe_2_O_3_, hematite, 99+%, Fischer Scientific, and TiO_2_, rutile, Sigma-Aldrich) with a BET surface area of 30 ± 1 m^2^/g, 80 ± 10 m^2^/g, and 31 ± 4 m^2^/g, respectively, were heated in an oven at 473 ± 1 K overnight to remove organic contaminants and then pressed onto one half of a tungsten grid (ca. 5 mg). The grid was then placed in the sample IR cell compartment, held by two stainless steel jaws. Following the preparation of the mineral sample and placement in the IR cell, the system was evacuated for 4 h using a turbomolecular pump. The mineral sample was subsequently exposed to 50% RH water vapor for 2 h to yield a hydroxylated terminated surface. Once hydroxylated, the system was evacuated for another 6 h to remove water vapor in the chamber. After evacuation, the sample was exposed to 10 mTorr of phenol vapor (99+%, Alfa Aser, Crystaline) for 20 min under dry conditions (RH < 1%), and adsorption of phenol was studied. Then the gas-phase phenol that remained in the chamber was evacuated for 2 h. The phenol sample was prepared by heating phenol crystals in a water bath at 333 K in which phenol vapor was prepared. For obtaining gas-phase phenol spectrum, FTIR spectra of the IR cell without the oxide surface present were collected for a cell with no phenol (background) and after introducing phenol vapor (sample).

Reactions of phenol with pre-nitrated oxide surfaces were also conducted. It is well established that mineral dust is often associated with nitrates in the atmosphere, especially aged mineral dust which can be transported from desert regions to more urban environments and remain present in the atmosphere for weeks.^48,74−78^ Oxide surfaces were first exposed to gas-phase HNO_3_ from the nitric acid vapor taken from a concentrated mixture of H_2_SO_4_ (∼96 w%): HNO_3_(∼70 w%) 3:1 ratio^79^ to form a nitrated oxide surfaces. A high HNO_3_ concentration was chosen to completely nitrate the surface. Oxide surfaces were first exposed to gas-phase HNO_3_ from the nitric acid vapor taken from a concentrated mixture of H_2_SO_4_ (∼96 w%): HNO_3_(∼70 w%) 3:1 ratio^79^ to form a nitrated oxide surfaces. A high HNO_3_ concentration was chosen to obtain a well-coated nitrated surface. Then, 10 mTorr of phenol was exposed to these surfaces to study the ON formation. In another set of experiments, adsorbed ON and oxidized products were exposed to 10 mTorr of SO_2_ (99+%, Sigma-Aldrich) for 30 min to study the formation of sulfated nitrophenols (SNP). FTIR spectra were collected over a 4 h period through both halves of the tungsten grid to monitor gas phase and particle phase. Following adsorption, the system was evacuated overnight. The experiments with HNO_3_ were conducted in a different experimental but very similar setup; instead of stainless steel a Teflon-coated cell was used. Relatively higher concentrations of phenol were used to obtain surface-product concentrations above the limit of detection for HRMS analysis.

Prior to and following the exposure to gases, single-beam spectra (250 scans) of the surface and gas phase were acquired using a resolution of 4 cm^–1^ over the spectral range extending from 600 to 4000 cm^–1^. Absorption spectra on oxide particles are reported as the difference in the oxide spectra before and after exposure to gases. Absorption bands due to gas-phase components, measured through the blank half of the tungsten grid, were subtracted to obtain FTIR spectra of adsorbed gases only.

### Ex Situ Analysis of Products Using High Resolution Mass Spectrometry

Organic products formed on oxide surfaces following reactions of phenol were analyzed using a direct-injection linear ion trap (ThermoFisher Orbitrap) high-resolution mass spectrometer (HRMS). Adsorbed products were extracted from the α-Fe_2_O_3_, TiO_2_, or SiO_2_ solid substrate using methanol (CH_3_OH, Fisher Scientific, HPLC grade) as the solvent. The sample vial, syringe, and all other glassware used in the transfer process were cleaned prior to use with methanol, and Milli-Q water (Millipore Sigma, 18.2 MΩ), and baked in an oven at 500 °C to further remove trace organics. Plastic vials used in sample preparation were sonicated in methanol for 60 min and washed thoroughly prior to using. All of the samples were stored at 250 K and analyzed within 48 h of collection.

HRMS analysis in both positive electrospray ionization (ESI) ([M + H]^+^ and [M + Na]^+^) and negative ESI modes ([M – H]^−^) was used. The heated electrospray ionization (HESI) source was operated at 100 °C. The ESI capillary was set to a voltage of 3.5 kV at 350 °C. The HESI-Orbitrap MS was calibrated prior to use. Mass spectra were acquired with a mass range of 50–2000 Da. Peaks with a mass tolerance of >4 ppm were rejected. For all samples, normalization level (NL), which is a direct measure of the counts per second lesser than 1.00 E7 was considered as below the limit of detection. Compositions were calculated with the following element ranges: 12C, 0–60; 1H, 0–150; 16O, 0–25; 14N, 0–5; 32S, 0–5; 23Na, 0–5; 48Ti, 0–5; 56Fe, 0–5; 28Si, 0–5. Tandem mass spectrometry (MS/MS) with a collision energy of 40 eV was used for structure determination. Additionally, standard phenol HRMS patterns were collected in different solvent systems and compared with the literature to rule out product formation during HRMS analysis. Methanol was then chosen as the solvent for these studies as it provided the best relative intensity.

### Reaction Thermodynamics and Vibrational Frequency Calculations

Spartan ’20 Version 1.1.4 (wavefunction Inc)^80,81^ was used to calculate the thermodynamics of the different reaction pathways as well as molecular vibrational frequencies of some of the surface products. Here, the geometries for adsorbed species were calculated with B3LYP, 6-311+G** level, and thermodynamic parameters for adsorbed species on α-Fe_2_O_3_ surfaces were calculated with density functional EDF2, 6-311+G** level. First, a small cluster of an α-Fe_2_O_3_ surface with 12 Fe atoms (one unit cell) was built, and each adsorbed species cluster was built on this surface.^82^ Vibrational frequencies for gas-phase species were calculated using a molecular mechanics model. All adsorbed species were considered to have monodentate adsorption in order to include more conformers. Molecular Mechanics Force Field (MMFF) was used as the force field. These parameters were chosen to reduce the computational cost while obtaining best accuracy and convergence for these calculations.

## Results and Discussion

### Adsorption of Phenol on Mineral Oxide Surfaces

**Figure 1:**
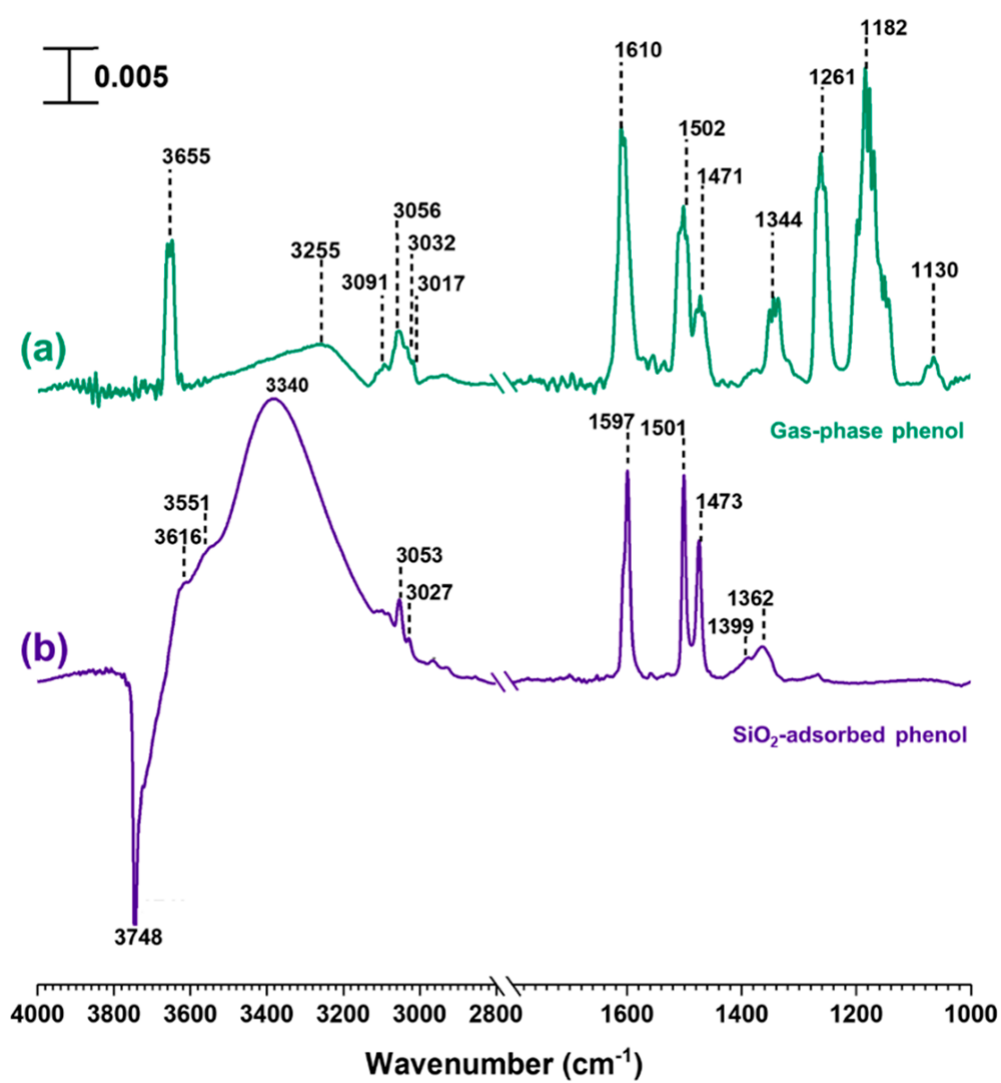
FTIR spectra of (a) gas-phase phenol and (b) SiO_2_ adsorbed phenol under dry conditions at a pressure of 10 mTorr. All the spectral features on SiO_2_ disappeared following desorption of phenol. The spectral regions from 1000 to 1800 cm^–1^ and 2800 to 4000 cm^–1^ are shown. The absorbance scale is also given in the top left corner.

The infrared spectrum of gas-phase phenol is shown in Figure 1. The spectrum collected at phenol pressure of 100 mTorr exhibits several peaks corresponding to aromatic ring vibrations as well as C–H and O–H bond vibrations. These include the O–H stretching mode at 3655 cm^–1^, aromatic C–H stretching modes ∼3000 to 3100 cm^–1^, and aromatic ring stretching and torsional modes at 1610, 1502, and 1471 cm^–1^.^83^ Additionally, the O–H bending mode at 1344 cm^–1^, the C–O stretching mode at 1261 cm^–1^, and C–H in-plane deformations at 1182, and 1130 cm^–1^ were also observed (see Table 1). The vibrational spectrum and vibrational frequencies of gas phase phenol can be compared to the spectrum of phenol adsorbed onto SiO_2_. SiO_2_ surfaces exposed to gas-phase phenol at 10 mTorr pressure under dry conditions show spectral features corresponding to adsorbed phenol. Additionally, a loss of surface silanol groups at 3748 cm^–1^ were seen suggesting the interaction of phenol with these Si–OH groups. Vibrational frequencies for adsorbed phenol were close to that of the gas-phase suggesting a relatively weak interaction. Moreover, almost all adsorbed phenol was removed from the surface via overnight evacuation, and HRMS analysis of solvent-extracted samples did not identify any peaks including those corresponding to phenol conclusively showing reversible adsorption of phenol on SiO_2_.

**Table 1:** FTIR Peak Assignments for Gas-Phase Phenol and Surface-Adsorbed Phenol^83,84^

IR peak assignment	gas-phase phenol	phenol + SiO_2_	phenol + α-Fe_2_O_3_	phenol + TiO_2_	phenol + nitrated α-Fe_2_O_3_	SO_2_ + phenol + nitrated α-Fe_2_O_3_
Surface Si–OH (loss)	--	3748	--	--	--	--
OH-stretching vibration of alcohols	3655	--	--	--	3685, 3661, 3628, 3609	--
Surface OH groups (loss)	--	--	3622	--	--	3674, 3620
O–H, hydrogen bonds	3255	3616, 3551, 3340	3520	--	--	3523
Aromatic C–H stretching	3091, 3056, 3032, 3017	3053, 3027	3068, 3030, 3012	3012	--	3079, 3037
C–H stretching	--	--	2959	--	2959, 2923, 2873, 2783	2960, 2926, 2888
Aromatic ring stretching and torsional modes	1610, 1502, 1471	1597, 1501, 1473	1594, 1549, 1531, 1490, 1427	1599, 1500, 1487, 1448	1581, 1481, 1447	1598, 1583, 1487, 1438
Adsorbed nitrate/nitrophenols	--	--	--	--	1634, 1581/1554	1544
Aliphatic C–H bending vibration	--	--	--	--	1375	1400
Ph–O–H bending vibration	1344	1362	1390, 1367, 1335	1396, 1360	1362	1355
Adsorbed sulfate	--	--	--	--	--	1333, 1314
Ph–C–O stretching vibration/ *adsorbed sulfate	1261	1261	1278, 1254, 1229	1278, 1232	1283, 1256	1276*, 1233*
Aliphatic C–O stretching vibration	--	--	1209	--	1201	--
C–H in-plane deformation	1182, 1130	--	--	--	--	--

**Figure 2:**
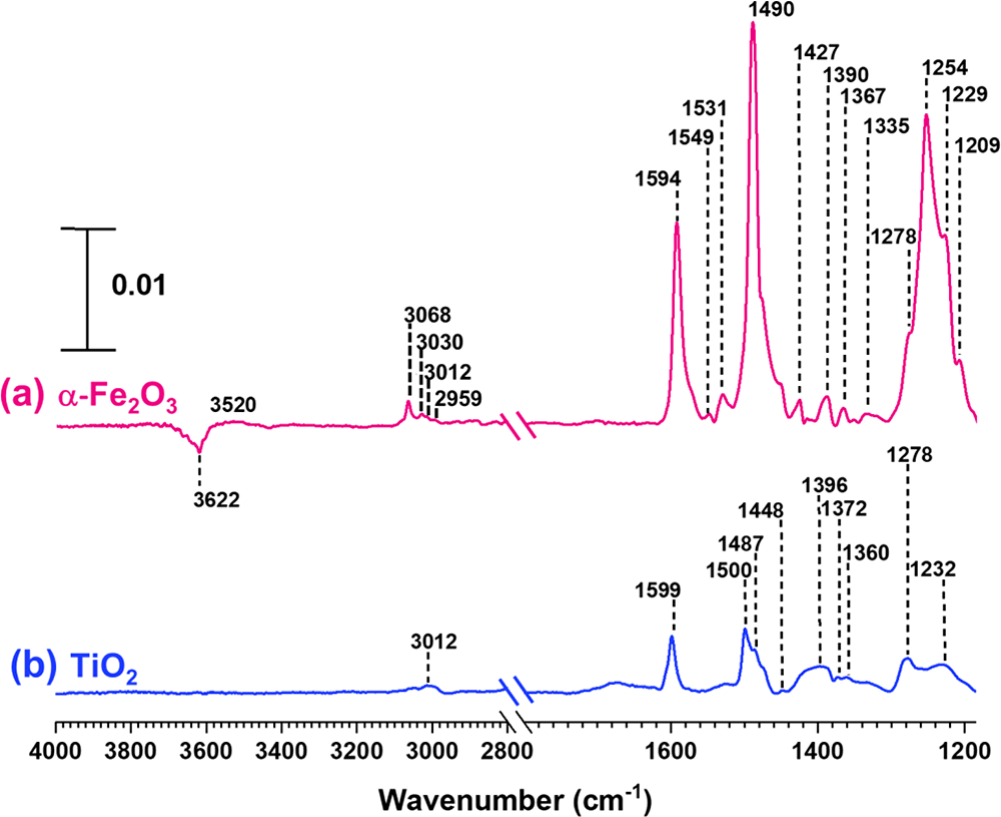
FTIR spectra of (a) α-Fe_2_O_3_ and (b) TiO_2_ following adsorption and evacuation of 10 mTorr phenol. These spectra are shown in the regions extending from 1150 to 1800 cm^–1^ and 2800 to 4000 cm^–1^. The absorbance scale is shown in the top left corner.

In contrast to SiO_2_, α-Fe_2_O_3_ surfaces exposed to phenol show new spectral features, suggesting the transformation of phenol to other compounds (Figure 2). Several peaks in the spectral region extending from 1427 to 1594 cm^–1^ were observed corresponding to aromatic ring vibrations. Moreover, a shift of these peaks to lower wavenumbers and additional peaks around 900 cm^–1^ indicate the poly-substitution of the aromatic ring.^83^ Additionally, a peak at 2959 cm^–1^ corresponding to aliphatic C–H bond stretching vibration and a peak at 1209 cm^–1^ corresponding to C–O bond vibration of aliphatic alcohols were observed. These new peaks suggest aromatic ring opening of phenol forming aliphatic compounds upon adsorption onto α-Fe_2_O_3_ surfaces.

TiO_2_, another reactive oxide found in mineral dust, similarly showed transformation of phenol upon adsorption. Although compared to α-Fe_2_O_3_, the surface spectrum following phenol adsorption onto TiO_2_ showed weaker spectral features. Nonetheless, evidence of formation of poly-substituted aromatic compounds such as several aromatic ring vibrations were observed. However, spectral features observed for on α-Fe_2_O_3_, i.e., including the presence of molecular species with aliphatic C–H bond stretching, and C–O bond vibration were not observed on TiO_2_ surfaces. To better understand the surface product formation from phenol adsorption, these products were solvent-extracted to methanol and analyzed via HRMS and MS/MS.

**Figure 3:**
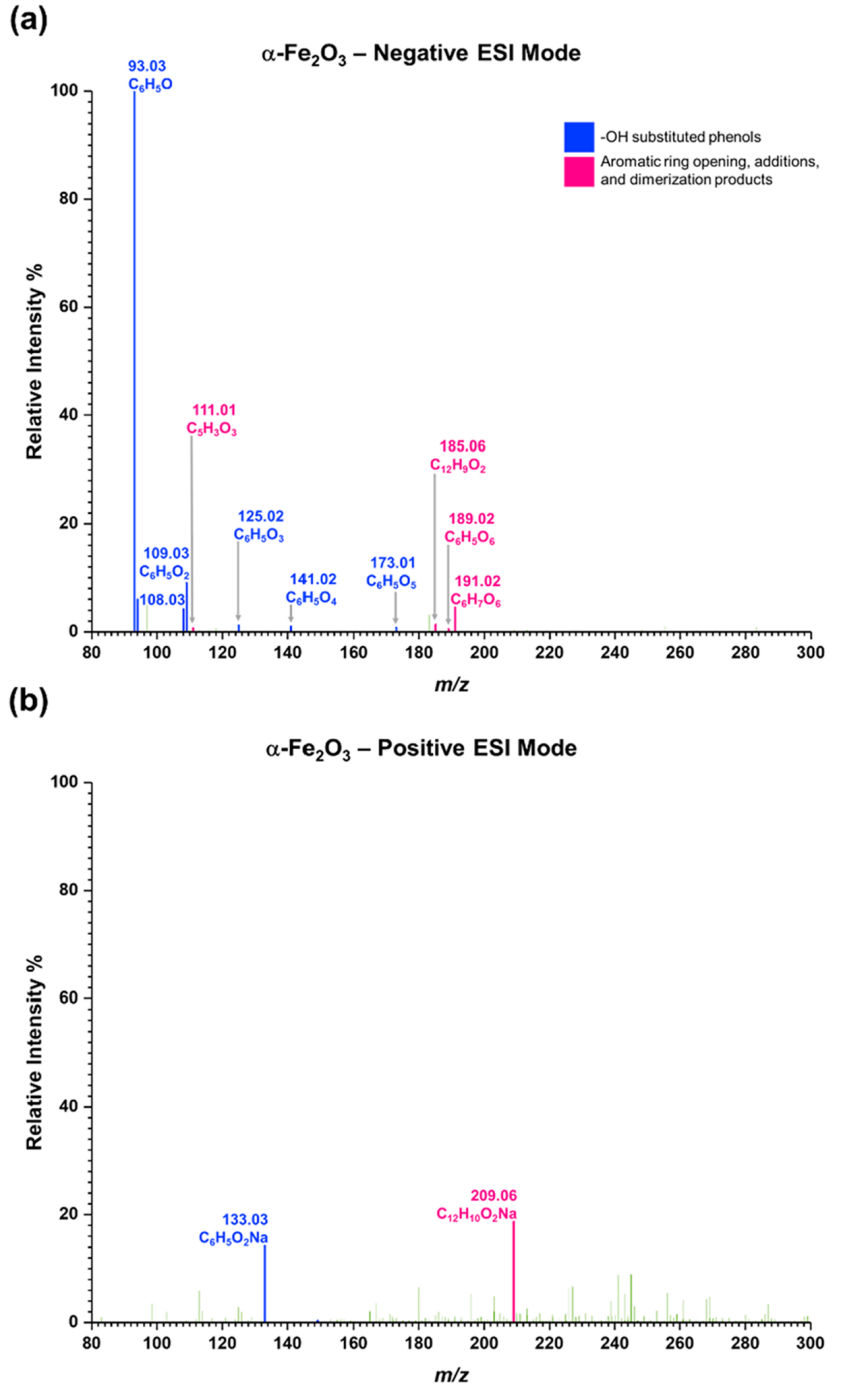
HRMS data for surface products formed upon adsorption of phenol on α-Fe_2_O_3_ surfaces under dry and dark conditions (a) negative ESI mode, (b) positive ESI mode.

Figure 3 shows the HRMS analysis for solvent-extracted surface products from phenol-exposed α-Fe_2_O_3_ surfaces in both negative ESI and positive ESI modes. As shown in Table 2, two main groups of surface products were identified following phenol adsorption onto α-Fe_2_O_3_ surfaces. These are (**A**) phenol and its OH substituted products and (**B**) aromatic ring opening and additional products. The primary OH substituted products observed on α-Fe_2_O_3_ surfaces are phenol (Compound **1**, C_6_H_5_O, *m/z* = 93.03, [−]), catechol and hydroquinone (Compound **2** and **3**, C_6_H_5_O_2_, *m/z* = 109.03, [−] and C_6_H_6_O_2_Na *m/z* = 133.03, [+]). Several other poly-substituted phenols were also observed on α-Fe_2_O_3_ surfaces. These are, Compound **4** and **5** (C_6_H_5_O_3_, *m/z* = 125.02, [−]), Compound **6** and **7** (C_6_H_5_O_4_, *m/z* = 141.02, [−]), and Compound **8** (C_6_H_5_O_6_, *m/z* = 173.01, [−]). Compounds **1**–**7** were also observed on TiO_2_ (Figure S1).

**Table 2:**
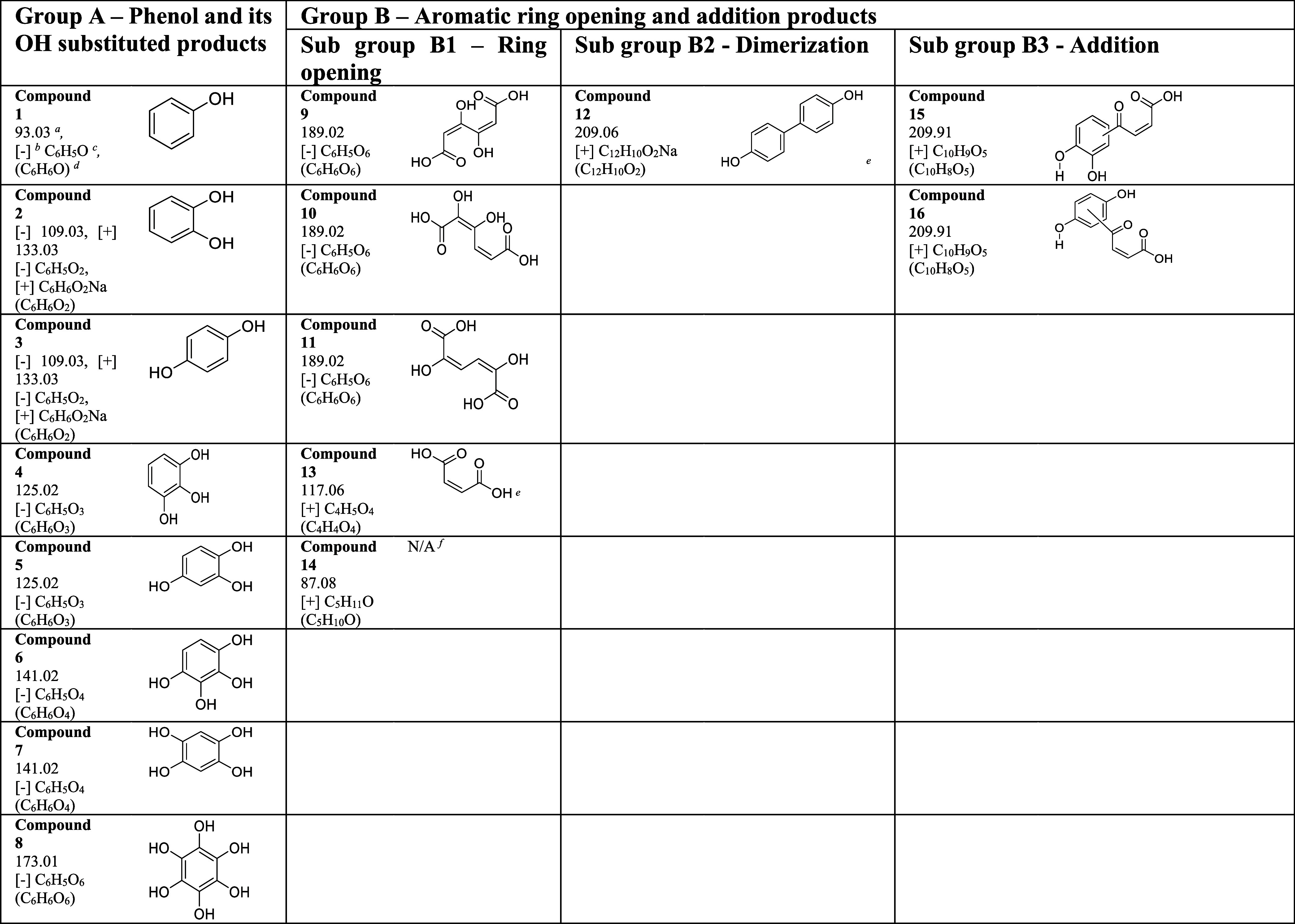
Identified Surface Products, Compounds **1** to **16**, from Phenol-Exposed α-Fe_2_O_3_ Surfaces Using HRMS Negative and Positive Electrospray Ionization (ESI) Modes

a *m/z*

b ESI mode.

c Observed formula.

d Molecular formula.

e Structure proposed based on MS/MS analysis, steric hindrance, stability, and conformer of parent molecule.

f Not available.

**Scheme 1:**
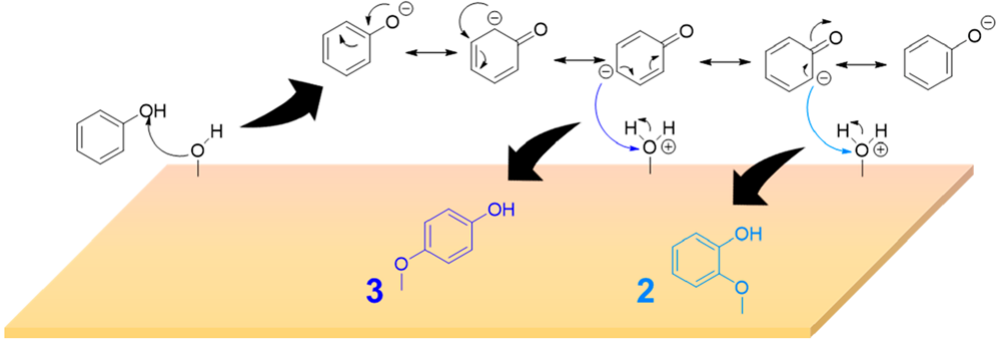
Proposed Formation Pathway for Compounds **2** and **3** from Phenol on α-Fe_2_O_3_ Surfaces *via* Interaction with Surface Hydroxyl Groups

Formation of OH substituted phenols can be due to the interaction with surface hydroxyl groups on α-Fe_2_O_3_ (Scheme 1). The OH group on phenol is ortho- para-directing due to the resonance stabilization. Electron-rich aromatic ring of phenol then reacts with positively charged oxygen atoms of surface hydroxyl groups (*now adsorbed water molecules*), thereby producing catechol (**2**) and hydroquinone (**3**). Further OH substitution to the aromatic ring is possible due to resonance activation, to form compounds **5**–**8** from either **2** or **3** as diphenols are more electron-dense than phenol. The ability to form oxidized polyphenols in the absence of photochemistry and/or strong oxidizing agents from adsorbed phenols underscores the potential importance of chemistry occurring on oxide surfaces in the absence of light.

**Scheme 2:**
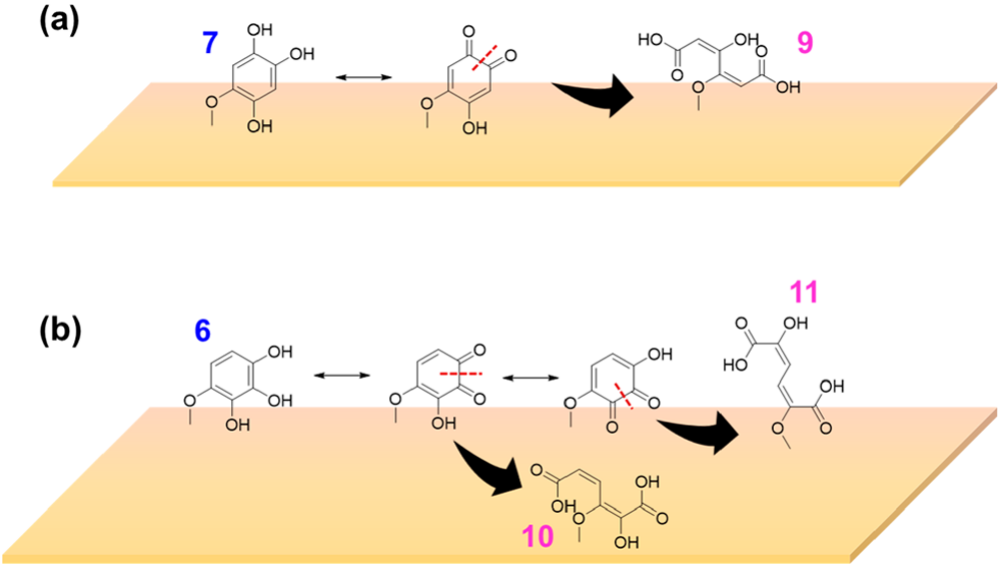
(a, b) Proposed Formation Pathways for Compounds **9**, **10**, and **11***via* Aromatic Ring Opening of Compounds **6** and **7** on α-Fe_2_O_3_ Surfaces

On α-Fe_2_O_3_ surfaces, products as a result of ring opening from compounds **6** and **7** were observed in negative ESI mode. These ring opening products include compounds **9**, **10**, and **11** (C_6_H_5_O_6_, *m/z* = 189.02). Due to the mass spectrometer internal chemistry, some of the C=C bonds in compounds **9**, **10**, and **11** are hydrogenated resulting in C_6_H_7_O_6_ at *m/z* = 191.02.^85^ These compounds produce a MS/MS fragment *m/z* = 111.01 for C_5_H_3_O_3_. However, **9**, **10**, and **11** produce a structurally similar fragment for C_5_H_3_O_3_, and thus we are unable to distinguish parent structures from one another. Formation of compounds **9** through **11** can be explained by a surface-mediated ring opening of compounds **6** & **7** (Scheme 2). Similar ring opening is previously observed with catechol and hydroquinone in the presence of radicals^14,37,86,87^ and with adsorbed 4-propyl-guaiacol and catechol during ozonolysis.^41,42^ During the oxidation of **6** and **7** to form **9** through **11**, Fe(III) on the α-Fe_2_O_3_ surface can be reduced to Fe(II). This redox chemistry underscores the importance of the different mineral reactivities; these ring opening products were not observed on TiO_2_ surfaces under similar conditions.

Additionally, on α-Fe_2_O_3_ surfaces, a phenol dimer (compound **12**, C_12_H_10_O_2_, *m/z* = 209.06, [+]) was observed. Dimerization of phenol in the presence of iron containing catalysts has been previously observed.^14,32^ Specifically, studies conducted on minerals, such as oligomerization of compounds with phenol moiety on Fe^3+^ saturated montmorillonite^88^ suggests that phenol oligomerization on Fe-containing minerals without photoirradiation is possible. Moreover, oligomerization of aminophenols in the presence of catalytic iron in aqueous environments were previously reported.^89^ The structure of **12** is proposed with a C–C bridge between the two aromatic rings instead of its isomer where a formation of C–O–C bridge occurs. This is because the resonance stabilization with the C–C bridge (Δ*G*° = −34.26 kJ/mol) is more stable than that of C–O–C bridge (Δ*G*° = 15.36 kJ/mol). The Gibbs free energies were directly derived from ChemDraw Professional 20.0.0.41, via the Joback Method.^14,32^

### Surface Reactions Leading to the Formation of Nitrophenols (NP) and Sulfated Nitrophenols (SNP)

Upon exposure of phenol to nitrated-α-Fe_2_O_3_ surfaces, i.e., surfaces first exposed to nitric acid vapor, spectral features for both aromatic and aliphatic compounds become apparent (Figure S2). Among these are prominent aliphatic C–H stretching vibrations between ∼2900 and 2700 cm^–1^, various alcohol O–H stretching frequencies ∼3600 cm^–1^, and C–H bending mode at 1375 cm^–1^. A peak for aliphatic C–O stretching vibration was observed at 1201 cm^–1^.^83^ Aromatic ring vibrations were observed ∼1481 and 1447 cm^–1^, whereas the Ph C–O stretching vibration were observed at 1256 cm^–1^, indicating the presence of aromatic alcohol compounds as well. Additionally, adsorbed nitrates were observed at 1581 cm^–1^. Therefore, the aromatic ring opening followed by formation of aliphatic organic compounds are apparent from this FTIR spectrum. Then, in a set of different experiments, these phenol-exposed nitrated-α-Fe_2_O_3_ surfaces were reacted with gas-phase SO_2_. Adsorbed sulfates can be seen on α-Fe_2_O_3_ surfaces ∼1300 and ∼1276 cm^–1^. Given the complexity of adsorbed surface products, many of the peaks cannot be assigned to one functional group type without compromising the accuracy. However, key features such as the presence of C–H stretching vibrations corresponding to both aromatic (∼3079, 3037 cm^–1^) and aliphatic (∼2900 cm^–1^) are present. Furthermore, unlike with phenol-exposed nitrated-α-Fe_2_O_3_ surfaces, here, a loss of surface hydroxyl groups was observed by a decrease in intensity of peaks at 3620 and 3674 cm^–1^, suggesting strong interactions between the surface OH groups and SO_2_(*g*).^90^ These surface products were solvent extracted and analyzed using HRMS to better understand and elucidate these surface products.

**Figure 4:**
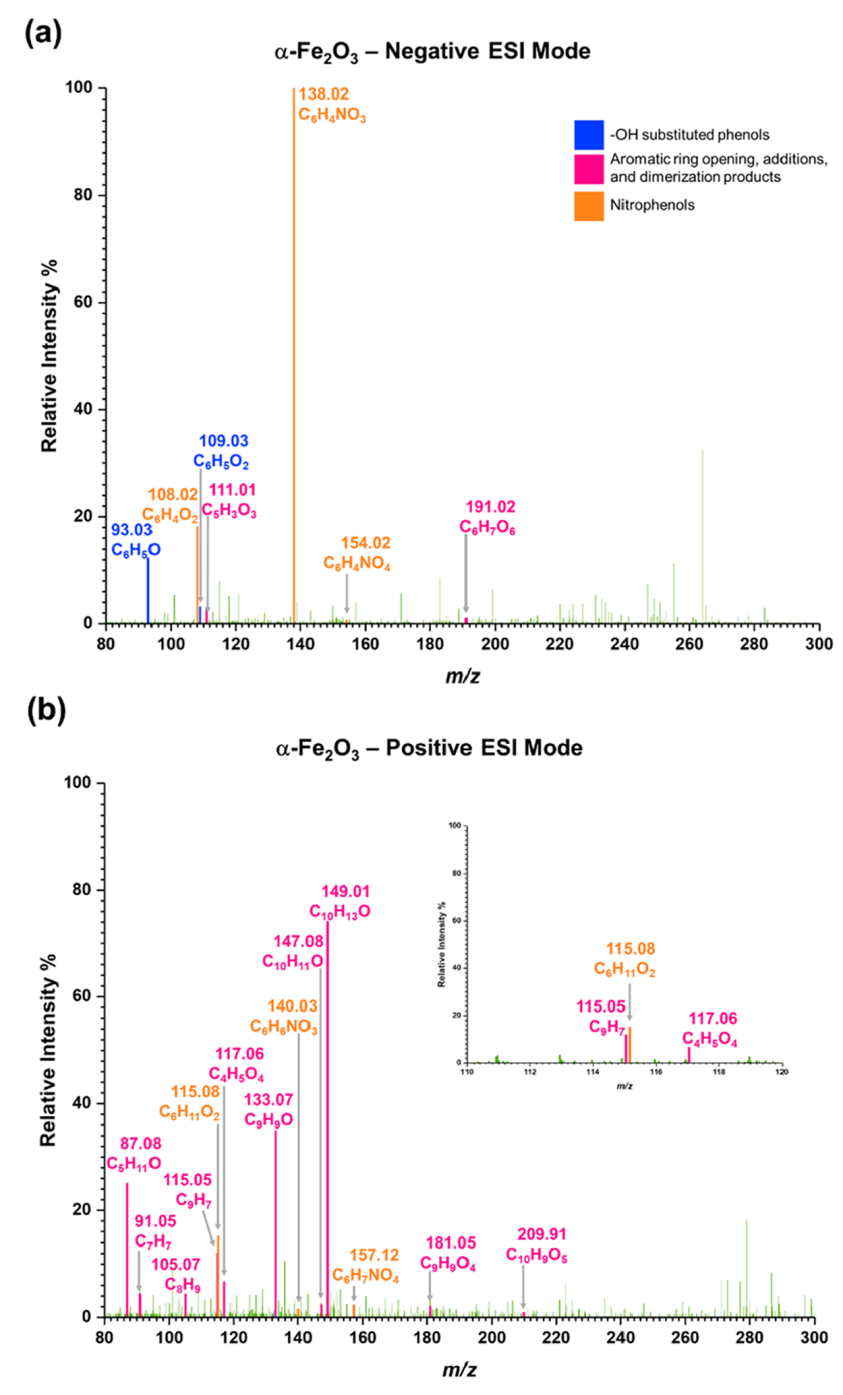
HRMS data for surface products formed upon reaction of phenol on nitrated α-Fe_2_O_3_ surfaces under dry and dark conditions (a) negative ESI mode, (b) positive ESI mode.

**Scheme 3:**
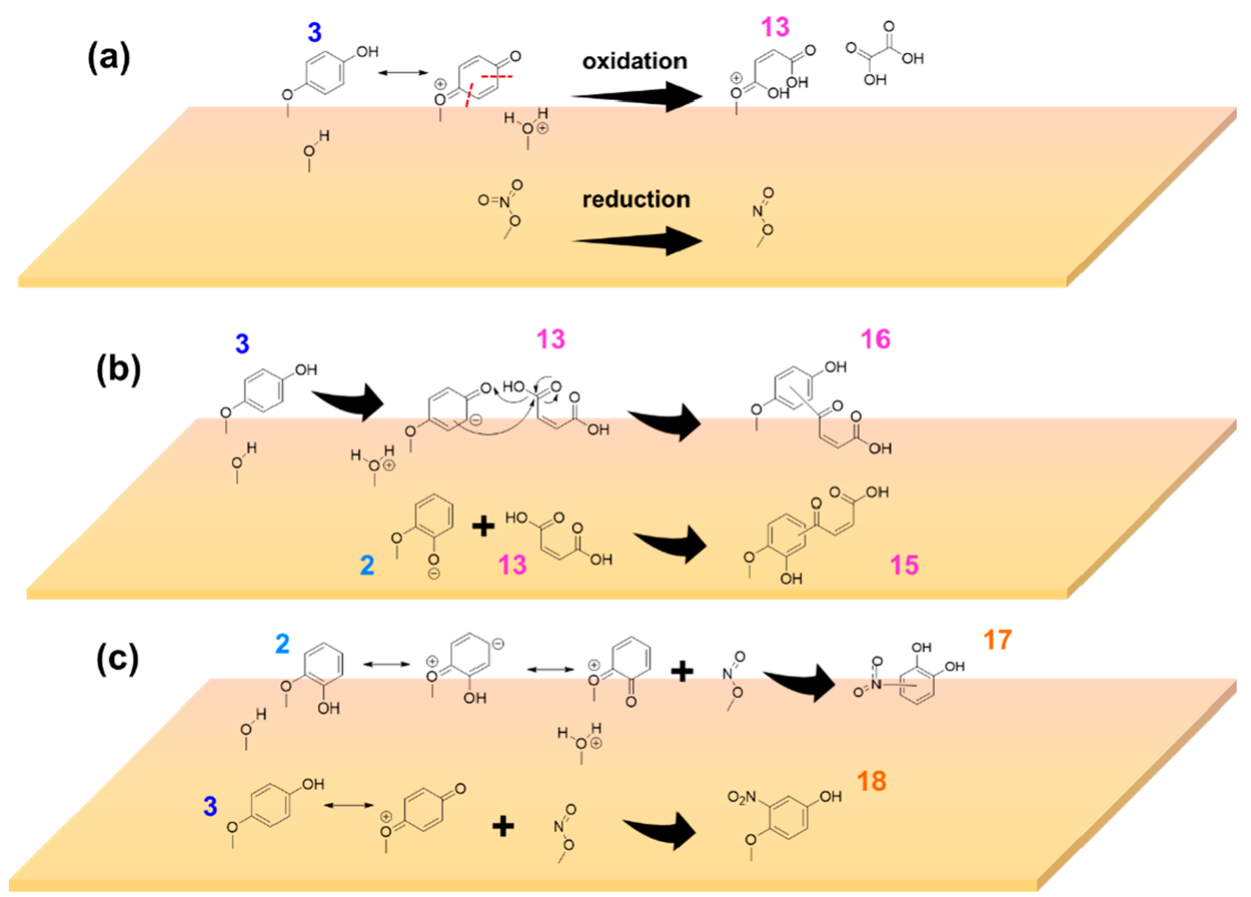
(a–c) Proposed Formation Pathways for Compounds **13**, **15**, **16**, **17**, and **18** Compound **13** forms *via* oxidation of **3** and reduction of adsorbed-nitrates to adsorbed-nitrites. Reactions between **3** and **2** with **13** yields **16** and **15** respectively. Reactions of **2** and **3** with adsorbed-nitrites form **17** and **18** respectively.

Figure 4 shows the HRMS analysis for solvent-extracted surface products from phenol-exposed nitrated-α-Fe_2_O_3_ surfaces in both negative and positive ESI modes. All compounds **1**–**11** were also observed in these experiments. An additional ring opening product (**13**) from **3** along with another compound **14** were observed, compound **13** for C_4_H_4_O_4_, *m/z* = 117.06, [+] and compound **14** for C_5_H_11_O, *m/z* = 87.08, [+]. Here, **3** can be resonance stabilized with para-quinone, which then oxidizes to maleic acid (**13**) and oxalic acid (not observed in HRMS) where adsorbed nitrates on α-Fe_2_O_3_ and lattice Fe(III) reduce to adsorbed nitrites and lattice Fe(II) respectively (Scheme 3a). Other surface mechanisms initiated by various adsorbed nitrogen species, such as NO_2_^+^, were not considered as previous research has found only formation of adsorbed nitrate on α-Fe_2_O_3_ surfaces when exposed to HNO_3_ vapor in dry and dark conditions.^64^ These additional surface products were not observed for nitrated-TiO_2_ surfaces indicating the important role surface Fe(III) plays here.

Peak deconvolution in the spectral region from 1100–1350 cm^–1^ for the FTIR spectra of evacuated phenol-exposed nitrated-α-Fe_2_O_3_ surfaces identified spectral features at 1310 and 1174 cm^–1^. These features were not present on phenol-exposed α-Fe_2_O_3_ surfaces, suggesting a minor nitrite formation on the nitrated surface (Figure S3). Adsorbed nitrites on oxide surfaces can be observed around 1230, 1313, 1192, and 1178 cm^–1^ depending on the oxide.^91^ Moreover, calculated vibrational frequencies for nitro bond vibrations of organonitrates, particularly nitrophenols, appear around 1628–1656 cm^–1^ distinguishing them from adsorbed nitrites. Additionally, the calculated Δ*G*° for the above surface-mediated redox reaction is −1.84 a.u., indicating the thermodynamic feasibility of such a surface reaction. As cis-trans isomers of **13** was not distinguished in our HRMS analysis; only the cis isomer (maleic acid) was considered. This is because due to the planar structure of hydroquinone, and the surface-adsorbed nature, formation of maleic acid is favored over fumaric acid. Additionally, maleic acid maybe formed from catechol oxidation where its ring-opening product *but*-2,4-dien-1,6-dicarboxylic acid further oxidizes in the presence of strong oxidizers. Adsorbed nitrates may facilitate this additional oxidation.^14^ The structure of **14** was not elucidated due to MS/MS fragmentation data falling below the limit of detection of the mass spectrometer utilized (*m/z* < 90).

Aromatic addition of **13** to either **2** (catechol) or **3** (hydroquinone) produces **15** and **16** (C_10_H_9_O_5_, *m/z* = 209.91, [+]) respectively (Scheme 3b). Here, adsorbed hydroquinone or catechol is electron-dense and can attack a nucleophilic carbon on maleic acid (**13**) forming structures **16** and **15** respectively. As the ring position of the maleic acid group is not determined, here it is shown as substituted to the aromatic ring.

In addition to these ring-opening products followed by aromatic substitution, two nitrophenols were identified on α-Fe_2_O_3_ surfaces. These are compounds **17** and **18** for (C_6_H_4_NO_4_, *m/z* = 154.02 [−]) (Table 3). The formation of **17** and **18** can be explained by the addition of either catechol or hydroquinone to surface-adsorbed nitrites formed from the above reaction in Scheme 3a. Formation of **17** from NO_2_^–^ and catechol has previously been observed in aqueous media and/or under photochemical conditions, and in ambient air.^16,34,86,92^ Moreover, comparing calculated vibrational frequencies for various nitrophenols with the deconvoluted spectra (Figure S3b), the peak at 1247 cm^–1^ corresponds to the aromatic C–O bond vibration of the alcohol group in the ortho position to the nitro group. The calculated bond vibration is positioned at 1242 cm^–1^ (from catechol, **2**) and 1251 cm^–1^ (from hydroquinone, **3**). However, the same bond vibration for an OH group at the meta position to the nitro group as in 4-nitrocatechol appears around 1269 cm^–1^, thus suggesting relatively higher abundance of 3-nitrocatechol (one possibility for **17** and 4-nitrocatechol being the other possibility) and 2-nitrohydroquinone (**18**). Here, the formation of surface-adsorbed nitrite was facilitated by the iron oxide surface and by the formation of hydroquinone from phenol underscoring the importance of mineral surfaces, their mineralogy, and their interactions with inorganic nitrogen species and organic species.

**Table 3:**
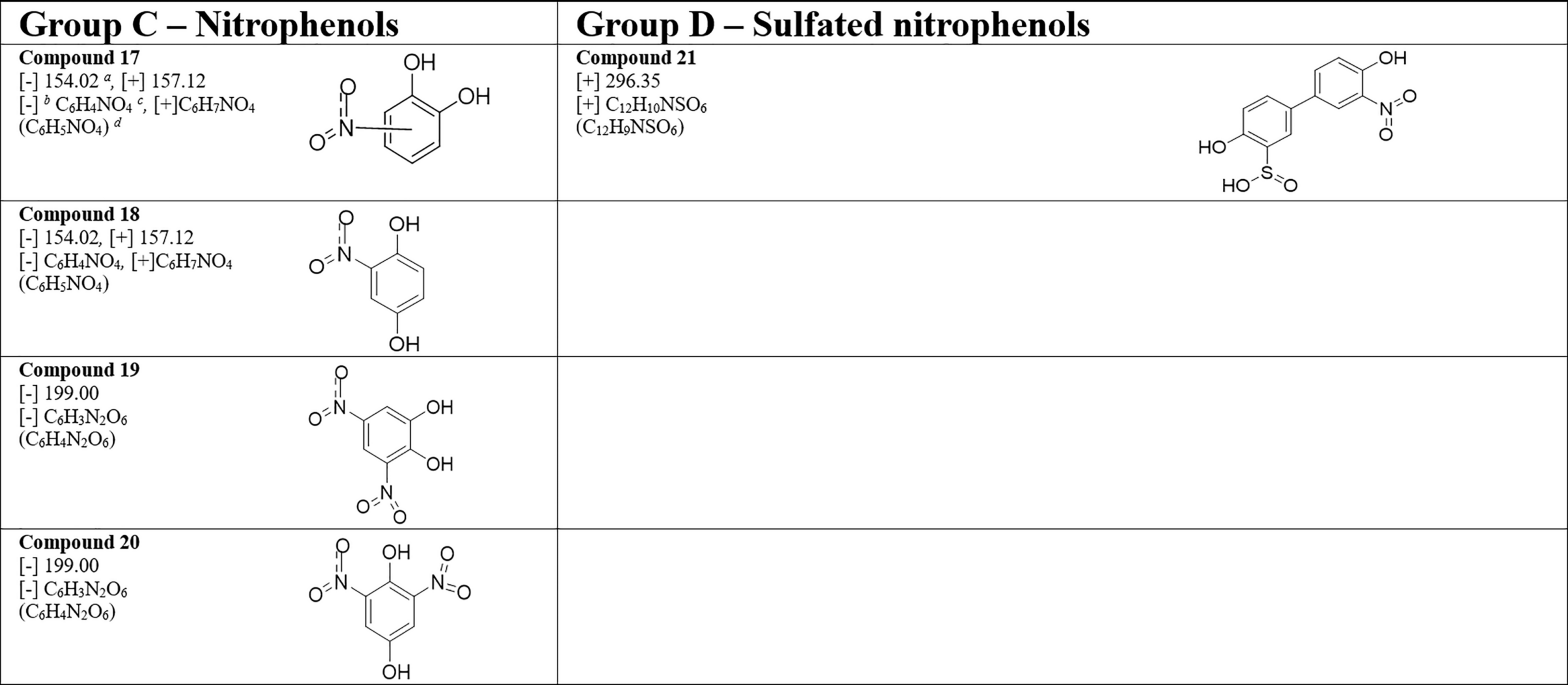
Identified Surface Products, Compounds **17** to **21**, from Phenol-Exposed Nitrated α-Fe_2_O_3_ Surfaces and from the Reaction of SO_2_ with Phenol-Exposed Nitrated-α-Fe_2_O_3_ Surfaces Using HRMS Negative and Positive Electron Spray Ionization (ESI) Modes

a *m/z*.

b ESI mode.

c Observed formula.

d Molecular formula.

**Figure 5:**
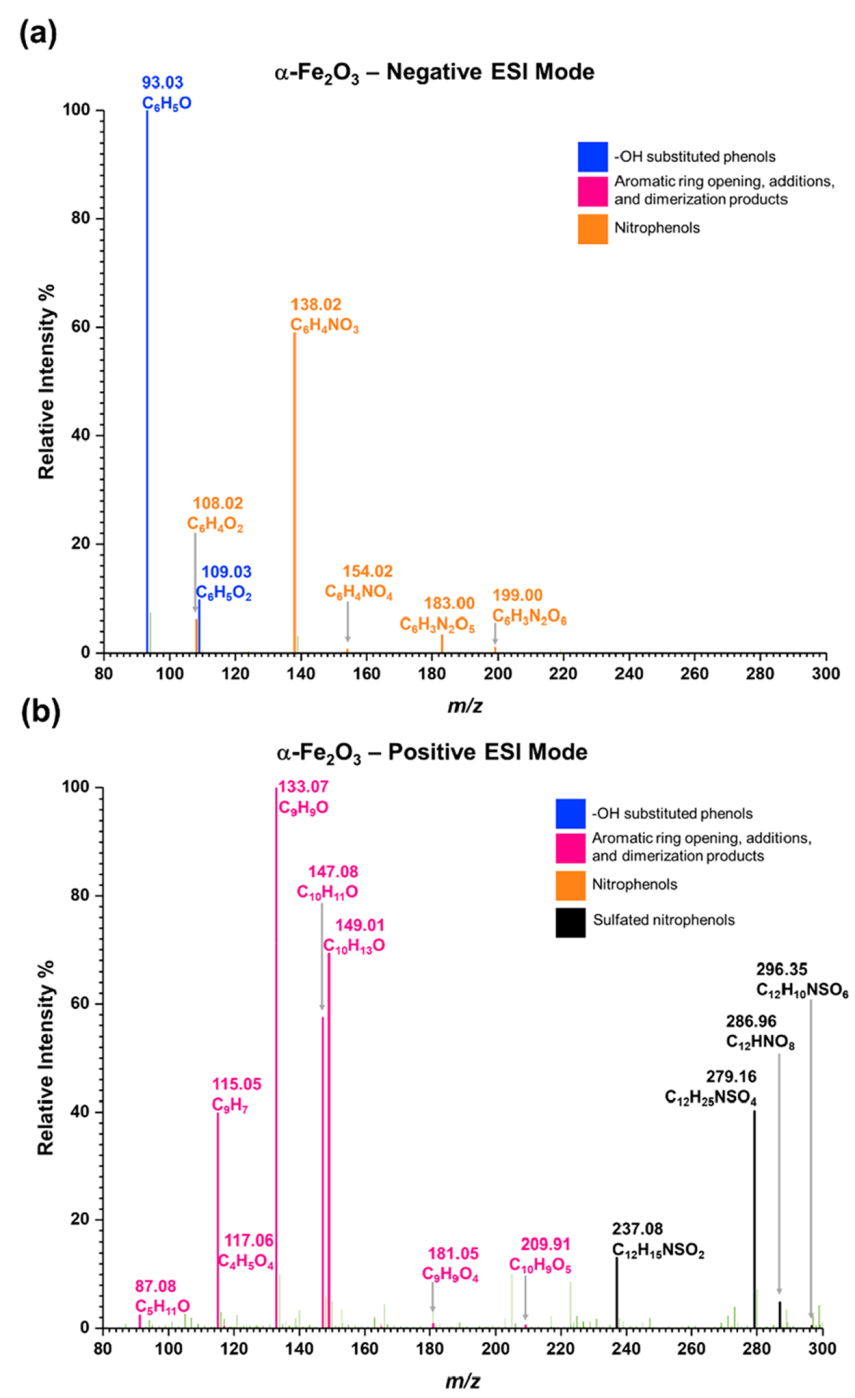
HRMS data of surface products formed upon reaction of SO_2_(*g*) on phenol-adsorbed nitrated-α-Fe_2_O_3_ surfaces under dry and dark conditions (a) negative ESI mode, (b) positive ESI mode.

Figure 5 shows the HRMS analysis for solvent-extracted surface products from the reaction of SO_2_(*g*) with phenol-exposed nitrated-α-Fe_2_O_3_ surfaces in both negative and positive ESI modes. All compounds **1**–**11** and **13**–**18** were observed in these experiments. A smaller quantity of a nitro-phenolic compound with two nitrate groups was observed upon reacting surface-adsorbed nitrophenols with SO_2_. This *m/z* peak was attributed to two possible structures each forming from **17** and **18**, **19** and **20** respectively for C_6_H_3_N_2_O_6_, *m/z* = 199.00 [−]. This extended nitration of catechol and hydroquinone as seen here can be attributed to the added formation of surface adsorbed nitrites by the oxidation of SO_2_(*g*). Other studies have shown nitrate-enhanced heterogeneous uptake of SO_2_ on mineral dust, where SO_2_ oxidizes to sulfate in the presence of adsorbed nitrate.^93−95^ In the current study, similar redox chemistry can occur between adsorbed nitrates and adsorbed SO_2_ forming adsorbed nitrites on the surface, thereby enabling extended nitration, similar to what is shown Scheme 3c. Moreover, different dinitrophenols and aromatic alcohols with multiple nitro groups are observed in ambient air.^17,96^ In addition to these nitrophenols, another compound derived from phenol dimer (**12**) was identified. Compound **21** for (C_12_H_10_NSO_6_, *m/z* = 296.35 [+]) can be formed from the nitration and sulfation of **12**.

## Conclusions and Atmospheric Implications

**Figure 6:**
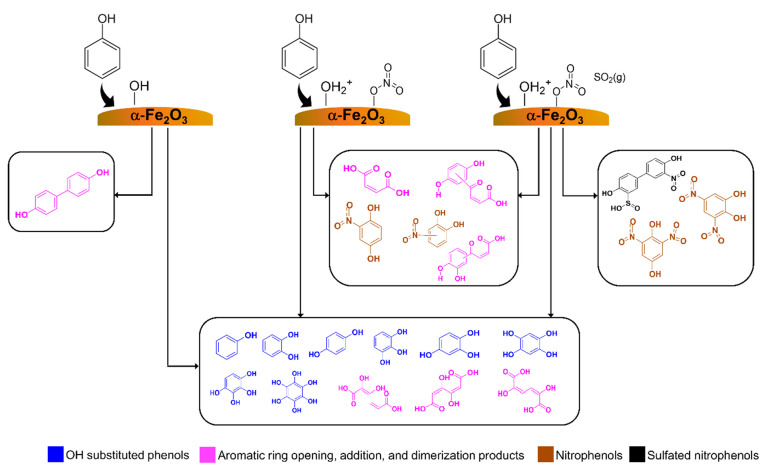
Solvent-extracted surface products identified via HRMS on α-Fe_2_O_3_ surfaces in each different systems under dry and dark conditions, (a) α-Fe_2_O_3_ upon exposure to phenol vapor, (b) pre-nitrated α-Fe_2_O_3_ upon exposure to phenol, and (c) where pre-nitrated α-Fe_2_O_3_ was exposed to phenol first, evacuated, and exposed to SO_2_ gas.

Phenol is shown to have complex surface chemistry on components of mineral dust aerosol. α-Fe_2_O_3_ is one of the most reactive surfaces, and reactions of phenol form OH substituted aromatic alcohols, primarily catechol and hydroquinone (Figure 6). There is also extended OH substitutions occurring to yield compounds with a stoichiometry of C_6_H_6_O_6_. Furthermore, some of the highly OH substituted aromatic alcohols undergo aromatic ring opening, thereby producing aliphatic oxidized SOA. This is especially important for α-Fe_2_O_3_ surfaces. The phenol dimer was also observed, suggesting phenol oligomerization on mineral dust surfaces. Although extended OH substitution was observed on TiO_2_ surfaces, there was no aromatic ring opening. These differences underscore the important role of mineralogy and different redox chemistry in heterogeneous reactions of mineral dust in the formation of SOA. Additionally, the reaction of phenol with nitrated-α-Fe_2_O_3_ surfaces yielded several nitro-phenolic compounds and other ring opening products. Hydroquinone form can be further oxidized forming maleic acid and oxalic acid, while reducing adsorbed nitrates and surface Fe(III) to adsorbed nitrites and surface Fe(II) respectively. These redox reaction products further undergo reactions with aromatic alcohols adsorbed on the surface to produce nitro-phenolic compounds and higher molar mass oxidized aromatic compounds. Most interesting are reactions of adsorbed nitro-phenolic compounds and nitrated-α-Fe_2_O_3_ surfaces with SO_2_ to produce additional nitro-phenolic compounds with two nitro-groups substituted to the aromatic ring. This is due to redox chemistry between SO_2_ and adsorbed nitrate leading to enhanced formation of adsorbed nitrites. These adsorbed nitrites facilitate the extended nitration of hydroquinone and catechol. Additionally, a sulfated nitrophenol derived from the phenol dimer was also observed in these reactions. These surface products stay surface-bound, thus increasing SOA formation. Additionally, these findings underscore the importance of particle mineralogy in heterogeneous mineral dust chemistry.

Overall, this study shows how mineral dust aerosol surfaces can provide a reactive surface whereby inorganic and organic gas-phase compounds when adsorbed can react to yield a wide range of compounds. These data show that surfaces can interact with gas-phase organics themselves and with surface adsorbed inorganic species to form SOAs. Interactions of gas-phase aromatic compounds with surfaces form either aromatic or aliphatic oxidized organic compounds, whereas interactions with adsorbed inorganics lead to the formation of nitrophenols and sulfated nitrophenols. Additionally, some of the SOAs observed in this study have been repeatedly observed in field studies, providing additional formation pathways aside from photochemical and/or gas-phase reactions to be understood. Additionally, the SOAs formed via these dark reactions can further react by undergoing photochemical reactions during the daytime, and/or interact with other oxidizers such as H_2_O_2_ and O_3_, thereby enhancing the molecular pool of compounds that can form on mineral dust surfaces. In the environment, VOCs such as phenol are ubiquitous. During dust transport and urban dust episodes, these VOCs and trace gas pollutants such as NO_2_, HNO_3_, and SO_2_ can interact with each other, forming more highly functional, less volatile compounds as shown here on Fe-containing mineral surfaces under dry conditions. As water plays an important role in heterogeneous chemistry,^97^ and particularly in the formation of substituted phenols such as aromatic alcohols and nitro-phenolic compounds, these are enhanced in aqueous environments,^14,16,33,38,40,42,53^ and thus it is important for future studies to address the role of adsorbed water on these different reaction pathways.

## Supporting Information Available

Three figures and one table including HRMS data of solvent-extracted surface products from phenol-exposed TiO_2_ surfaces (Figure S1); FTIR spectra of evacuated surfaces of phenol reaction with nitrated α-Fe_2_O_3_ and reaction of SO_2_(g) with adsorbed phenol on nitrated α-Fe_2_O_3_ surfaces (Figure S2); deconvoluted FTIR spectra of phenol-exposed α-Fe_2_O_3_ and nitrated-α-Fe_2_O_3_ surfaces (Figure S3); and MS/MS analysis of parent peaks for the identified compounds (Table S1) (PDF)
