# Supplementary material for: Heterogeneous Reactions of Phenol on Different Components of Mineral Dust Aerosol: Formation of Oxidized Organic and Nitro-Phenolic Compounds
Source: ACS EST Air. 2024 Feb 23;1(4):259–72. doi: 10.1021/acsestair.3c00042 (PMC11019555; doi:10.1021/acsestair.3c00042)
Supplement: Supplementary file 1 — ea3c00042_si_001.pdf [file ea3c00042_si_001.pdf]

Supporting Information
for
Heterogeneous Reactions of Phenol on Different Components of Mineral Dust Aerosol: Formation of Oxidized Organic and Nitro-Phenolic Compounds

Eshani Hettiarachchi[1] and Vicki H. Grassian[1, *]

[1] Department of Chemistry and Biochemistry, University of California San Diego, 9500 Gilman Drive, La Jolla, California 92093, United States

The Supporting Information (SI) contains three figures and one table.

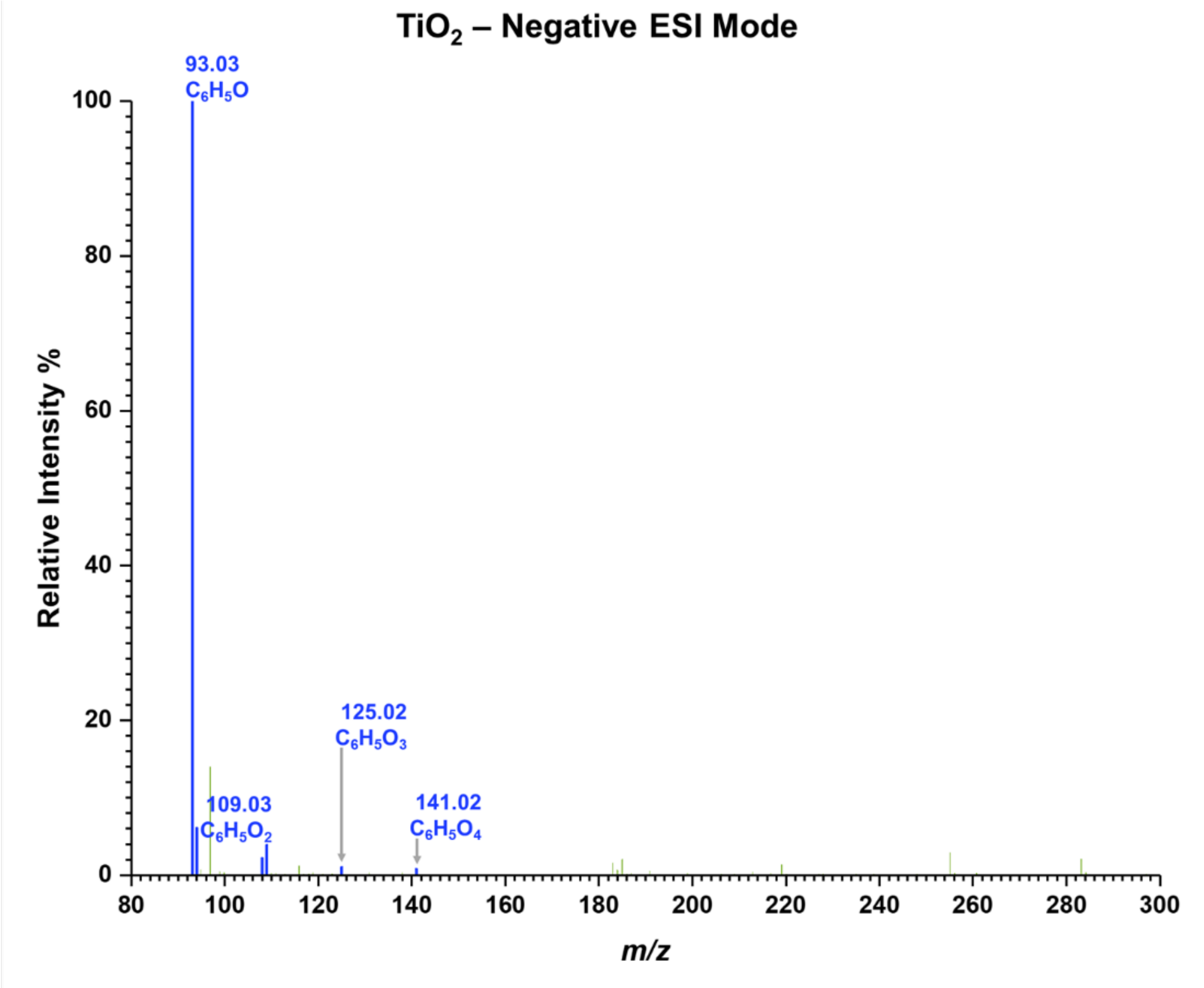

Figure S1: HRMS patterns of surface products formed upon adsorption of phenol on TiO_2 surfaces under dry and dark conditions in negative ESI mode.

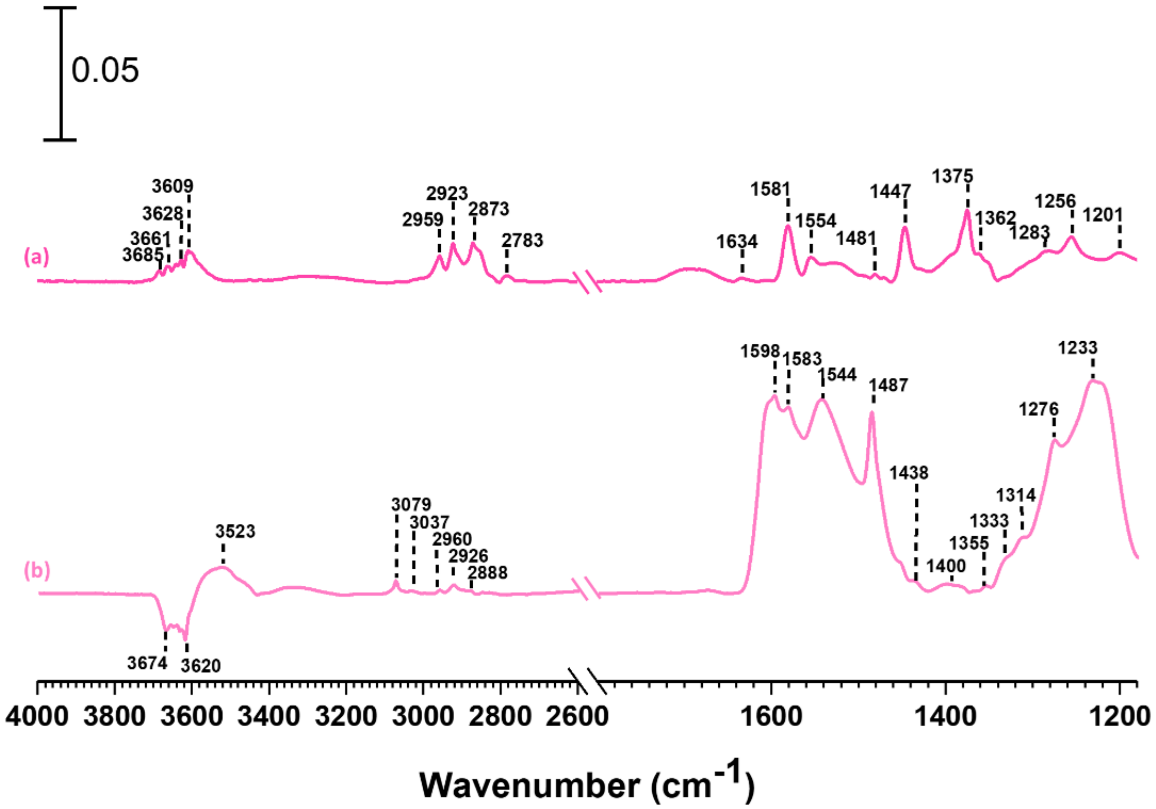

Figure S2: FTIR spectra of evacuated surfaces of (a) phenol (10 mTorr) reaction with nitrated α-Fe_2O_3 and (b) reaction of SO_2(g) (10 mTorr) with adsorbed phenol on nitrated α-Fe_2O_3 surfaces under dry conditions in the spectral regions from 1150 to 1800 cm^{-1} and 2800 to 4000 cm^{-1}. The absorption scale is shown in the top left corner.

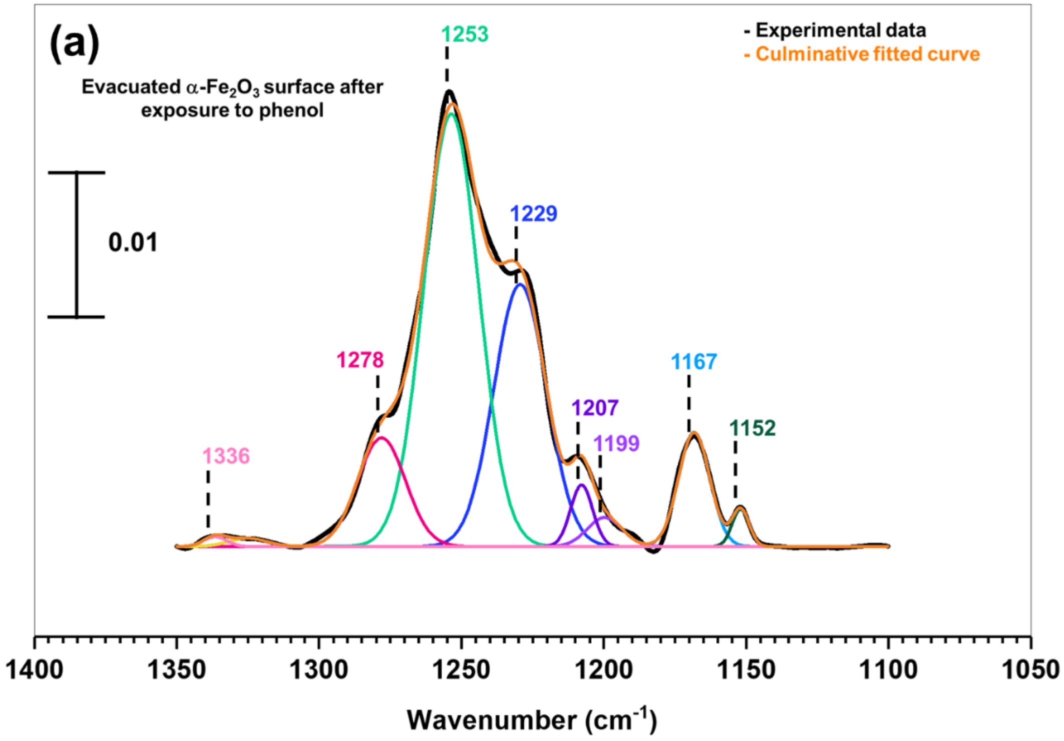

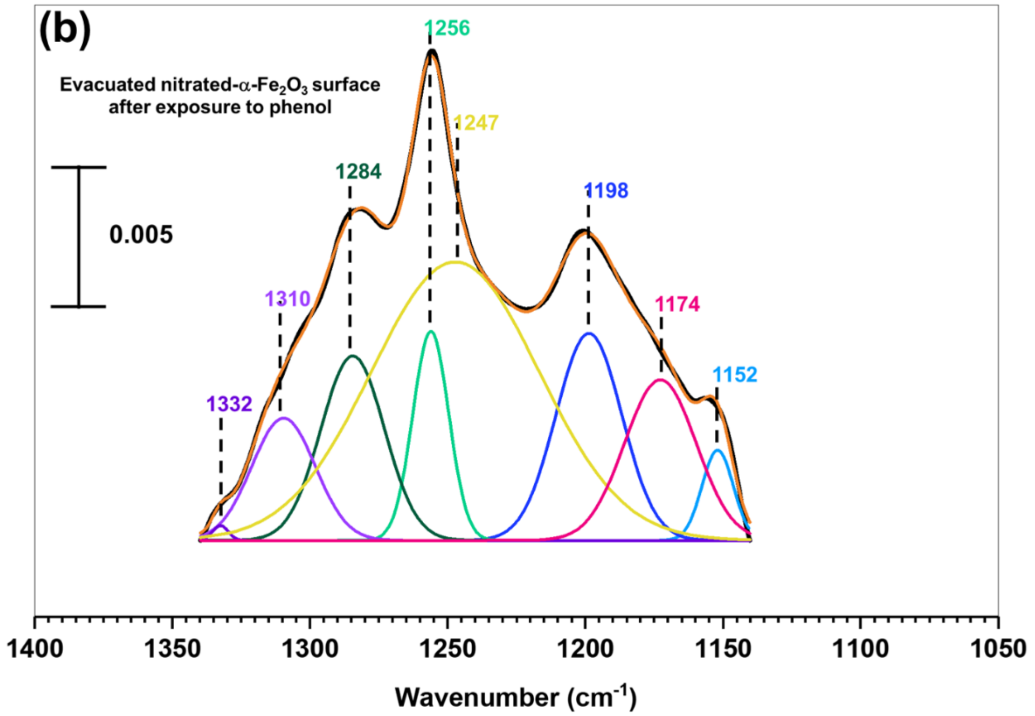

Figure S3: Deconvoluted spectra of evacuated (a) α-Fe_2O_3 and (b) nitrated α-Fe_2O_3 surfaces after exposure to phenol. Peak deconvolution was performed with Origin Pro 2017b; Gaussian function, straight baseline with auto baseline subtraction, with Quadratic-Savitzky-Golay method for smoothing, with 2nd derivative for searching hidden peaks were utilized.

Table S1: MS/MS Analysis for Parent Peaks of Identified Compounds

ID	Parent Peak, Observed Formula, ESI Mode	Fragment	Formula
**2 and 3**	109.03, $C_6H_5O_2$, Neg	75.02	C_6H_4
**9, 10, 11**	189.02, $C_6H_5O_6$, Neg	111.01	$C_5H_3O_3$
	191.02, $C_6H_7O_3$, Neg	189.02	$C_6H_5O_6$
**13**	117.06, $C_4H_5O_4$, Pos	115.05	C_9H_7
		105.07	C_8H_9
		91.05	C_7H_7
**15 and 16**	209.91, $C_{10}H_9O_5$, Pos	181.05	$C_9H_9O_4$
		149.01	$C_{10}H_{13}O$
		147.08	$C_{10}H_{11}O$
**17 and 18**	154.02, $C_6H_4NO_4$, Neg	138.02	$C_6H_4NO_3$
		108.02	$C_6H_4O_2$
	157.12, $C_6H_7NO_4$, Pos	140.03	$C_6H_6NO_3$
		115.08	$C_6H_{11}O_2$
**19 and 20**	199.00, $C_6H_3N_2O_6$, Neg	183.00	$C_6H_3N_2O_5$
**21**	296.35, $C_{12}H_{10}NSO_6$, Pos	286.96	$C_{12}HNO_8$
		279.16	$C_{12}H_{25}NSO_4$
		237.08	$C_{12}H_{15}NSO_8$
